# Case Report: A case of tuberculous empyema causing rupture of the diaphragm was misdiagnosed as diaphragmatic hernia

**DOI:** 10.3389/fmed.2025.1621689

**Published:** 2025-08-13

**Authors:** Tao Liu, Ting Yi Zhou, Guang Yu Li, Jing Feng Xu

**Affiliations:** Department of Thoracic Surgery, Guiyang Public Health Clinical Center, Guiyang, China

**Keywords:** tuberculous, empyema, diaphragm rupture, subcapsular liver, thoracic surgery

## Abstract

Tuberculous empyema (TE) is a chronic active infection caused by *Mycobacterium tuberculosis* that invades the pleural cavity. Initially, fluid accumulates in the pleural space, followed by an influx of neutrophils, which gradually develops into purulent fluid. This process can eventually lead to pleural thickening and calcification, restricting lung expansion and impairing lung function. Additionally, empyema can extend outward through weaknesses in the chest wall, forming abscesses in the soft tissues outside the thoracic cavity. The combination of anti-tuberculosis medications and surgical intervention is a crucial treatment approach for tuberculous empyema. We report a case of tuberculous empyema that invaded the diaphragm, resulting in diaphragmatic rupture and the formation of a subcapsular liver abscess, which was initially misdiagnosed as a diaphragmatic hernia. The patient showed significant improvement and was discharged following surgical treatment.

## Introduction

Tuberculosis (TB) remains a major public health challenge globally. As reported in the Global Tuberculosis Report 2024, TB is the second most prevalent infectious disease, following only by novel coronavirus infections. It is also the second leading cause of death from a single infectious agent worldwide, ranking thirteenth among all causes of death (1).

Tuberculous empyema frequently arises due to the rupture of pulmonary tuberculosis cavities or subpleural caseous lesions, thereby causing pleural infection. Additionally, it may result from the direct extension of paravertebral abscesses secondary to spinal tuberculosis, the dissemination of infection via thoracic lymph nodes or subdiaphragmatic lesions, or hematogenous spread (2). According to the European Association of Cardio-Thoracic Surgery (EACTS), empyema is categorized into three progressive stages: the parapneumonic effusion stage (stage 1), the purulent fibrinous stage (stage 2), and the chronic organizing stage (stage 3) (3, 4). In certain cases, long-standing non-resolving exudative pleural effusion can progress to empyema. Chronic empyema is characterized by purulent inflammation, pleural thickening, substantial fibrosis, and the proliferation of granulation tissue with purulent characteristics. In its advanced stages, it may lead to restriction of the affected lung, thoracic collapse with restrictive features, and significant impairment of cardiac and pulmonary functions (5).

Untreated or inadequately treated tuberculous empyema may result in pleurocutaneous fistula, chest wall mass, and rib or bone destruction (6). The empyema can extend outward through the weak areas of the parietal pleura and chest wall, leading to the formation of abscesses in the soft tissues outside the thoracic cavity (7). There are limited reports regarding empyema causing diaphragmatic rupture. We present a case of tuberculous empyema that invaded the diaphragm, resulting in diaphragmatic rupture and the formation of a subcapsular liver abscess, which was initially misdiagnosed as a diaphragmatic hernia. Following surgical intervention, the patient demonstrated significant improvement and was subsequently discharged.

### Case information

**Figure 1:**
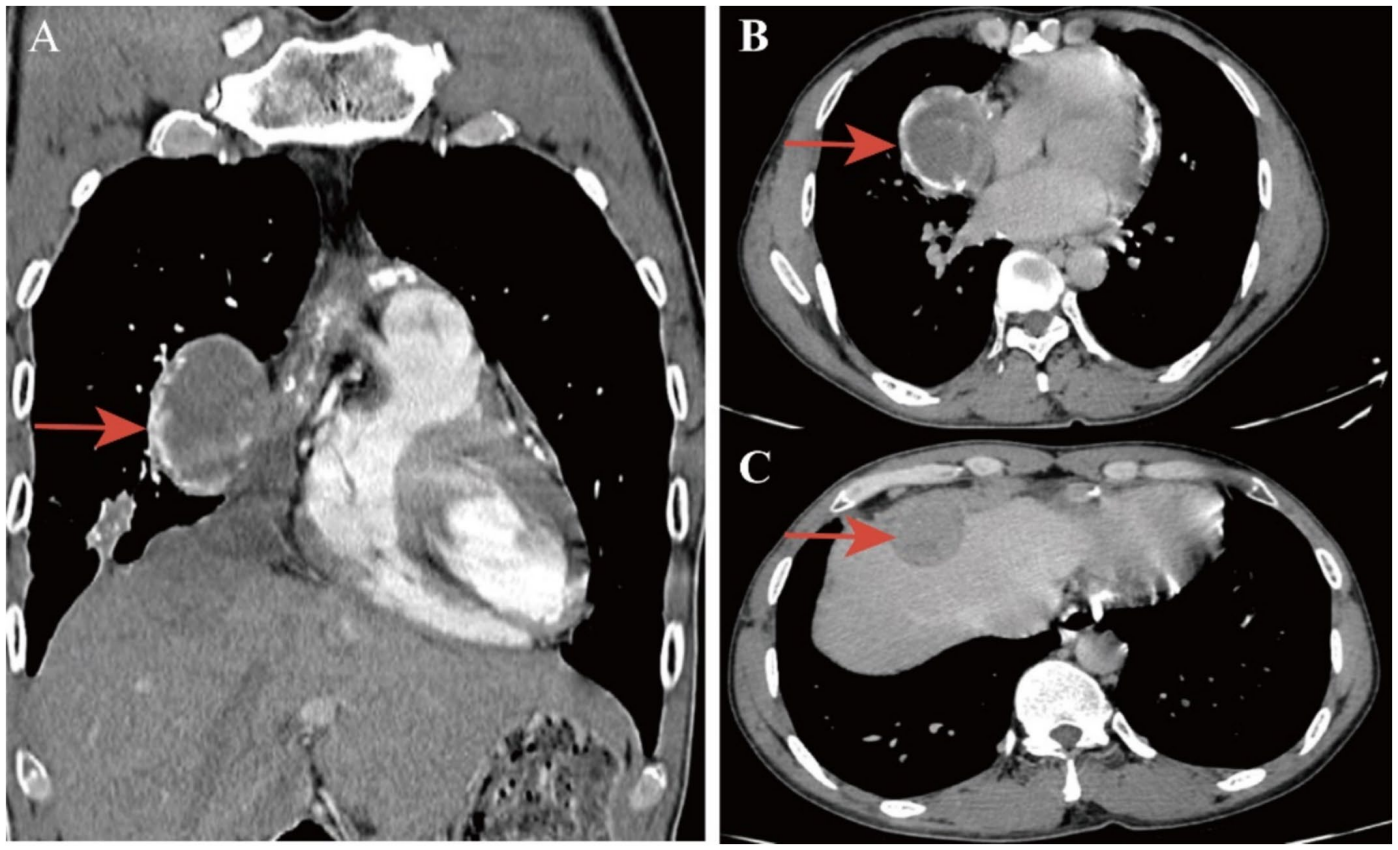
Enhanced chest CT showing lesions in the right mediastinal paravertebral and right cardiophrenic angle regions [**(A)** Coronal section, **(B,C)** Transverse section. Red arrows indicate mediastinal and liver capsule abscesses].

The patient is a 38-year-old male who has been living in Guizhou Province, an area with a high incidence of tuberculosis. Three months before admission, the patient reported experiencing postprandial abdominal distension and pain of unknown origin, accompanied by cough, chills, and fever, no nausea, vomiting, diarrhea, melena, chest tightness, palpitations, dyspnea, or any other discomfort. Following the diagnosis of tuberculosis, the patient was treated with a standard quadruple regimen of Isoniazid, Rifampicin, Pyrazinamide, and Ethambutol (HRZE) for anti-tuberculosis therapy. The patient’s symptoms of abdominal pain and distension improved significantly. However, one and a half months after initiating the medication, the patient discontinued the treatment on his own, then readmitted to the hospital due to recurrent abdominal pain and distension after meals. Physical examination revealed a flat abdomen without visible gastrointestinal contours or peristaltic waves. Tenderness was noted in the right upper quadrant, but there was no rebound tenderness or muscle rigidity. The liver and spleen were not palpably enlarged. There was also no shifting dullness. Chest and abdominal contrast-enhanced computed tomography (CT) demonstrated multiple lesions in both lungs, suggestive of secondary pulmonary tuberculosis with caseous pneumonia in the right middle lobe. Lesions in the right mediastinal paravertebral and right cardiophrenic angle regions, likely representing tuberculous pleurisy with diaphragmatic hernia formation and possible subcapsular liver abscess. Multiple calcified and partially enlarged mediastinal lymph nodes. Thickened and calcified pericardium, indicative of tuberculous involvement. Bilateral pleural thickening (Figure 1). Echocardiographic findings of the heart show mild tricuspid regurgitation. Reduced left ventricular diastolic function. Laboratory tests showed WBC count of 4.67×10^9/L, ESR of 20 mm/h, ALT of 19.2 U/L, AST of 17.7 U/L, ALB of 45.7 g/L, TBIL of 11.1 umol/L, DBIL of 3.6 umol/L, and IBIL of 7.5 umol/L. Serological tests for HBV, HCV, and HIV were negative. Acid-fast staining of stool and sputum smears was negative. The sputum *mycobacterium tuberculosis* (Mtb) DNA, *nontuberculous mycobacterial*-DNA, and GeneXpert MTB/RIF results were all negative.

### Clinical considerations and diagnostic and treatment measures

**Figure 2:**
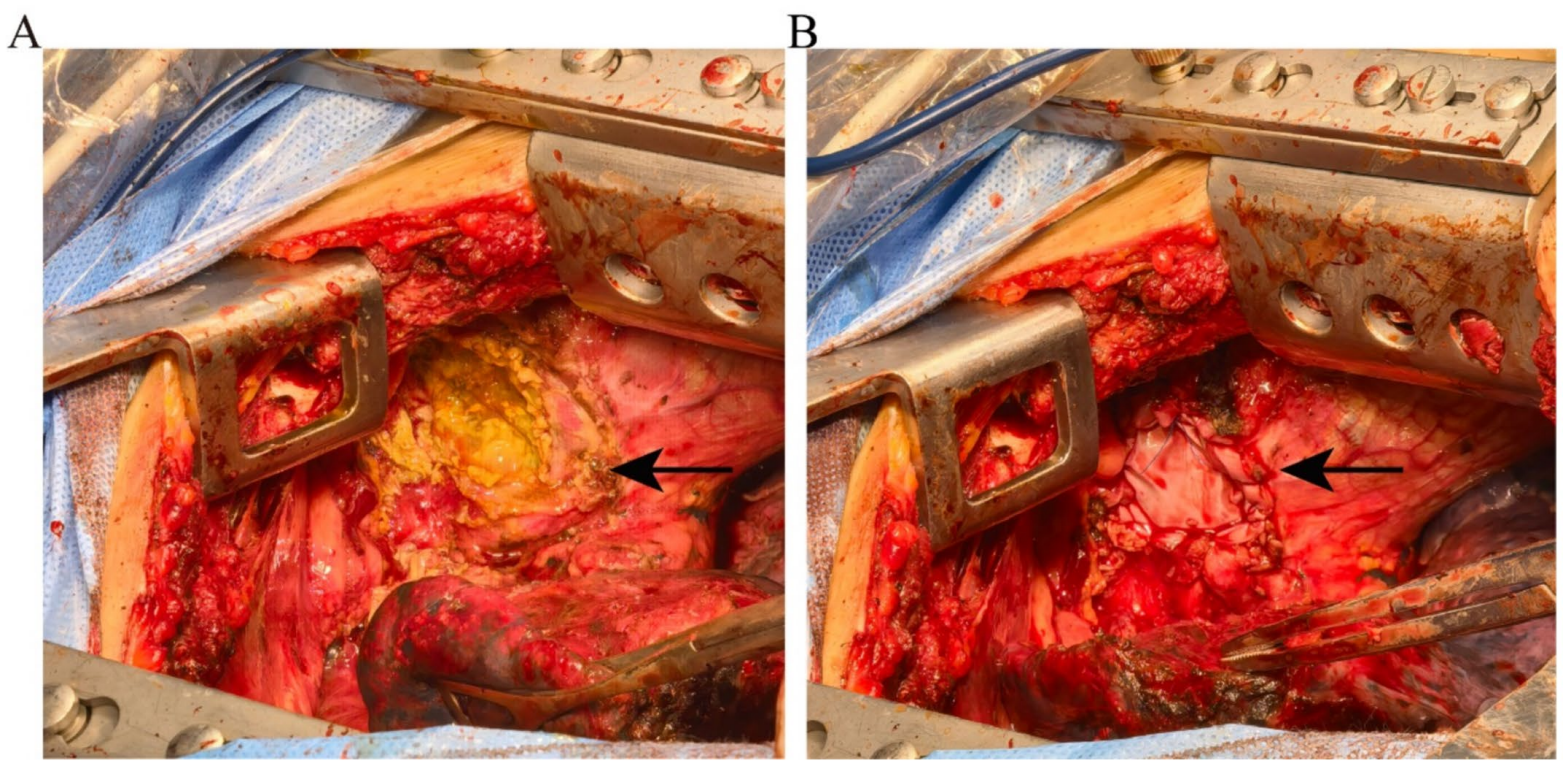
**(A)** Intraoperative images showing the empyema invading the subcapsular liver through the diaphragm. **(B)** The diaphragmatic rupture was repaired using a patch (the arrow indicates the diaphragm rupture).

**Figure 3:**
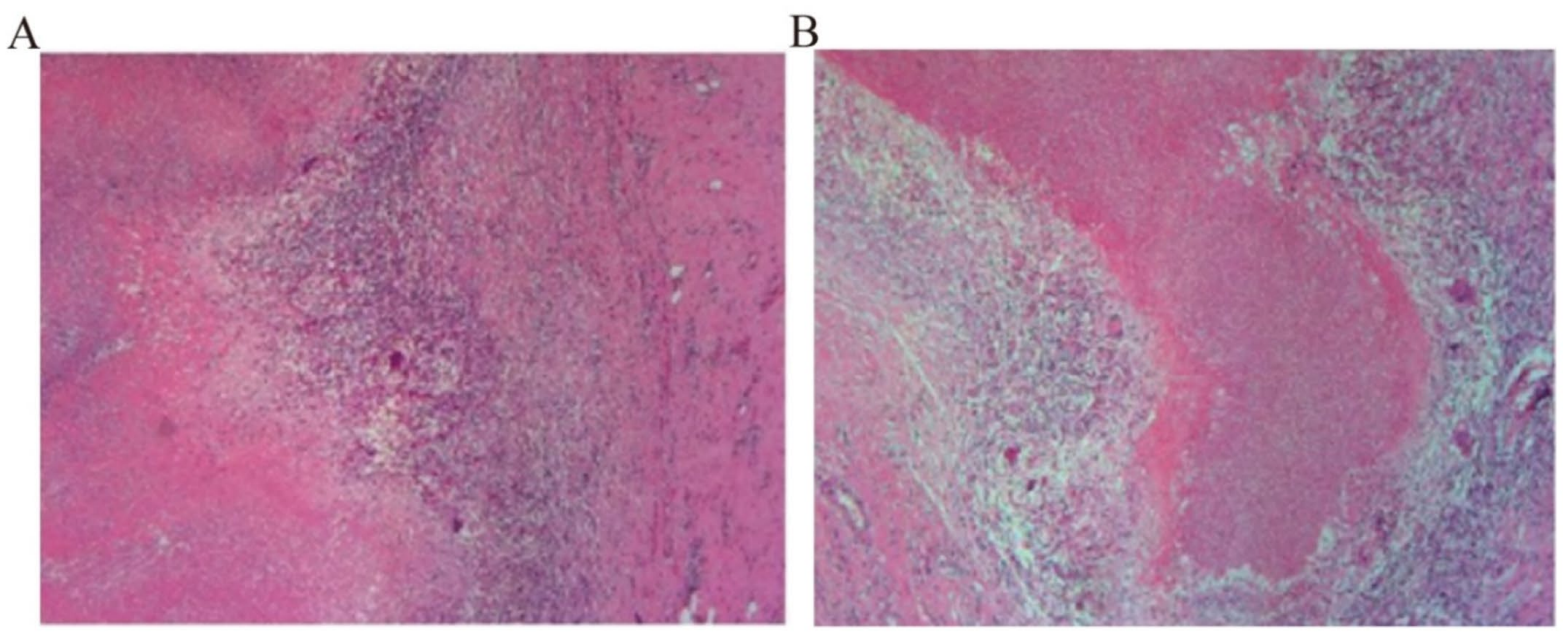
**(A,B)** Postoperative pathology of necrotic tissue of the mediastinum and diaphragm indicated necrotizing granulomatous inflammation.

The patient had a definite history of tuberculosis. The abdominal pain improved after the administration of oral anti-tuberculosis medications. However, the symptoms recurred following the patient’s self-discontinuation of the treatment. Physical examination did not reveal any signs suggestive of abdominal tuberculosis. Chest and abdominal contrast-enhanced computed tomography scans demonstrated lesions in the right paravertebral mediastinum and the right cardiophrenic angle region. It is suspected that the patient may have a combination of tuberculous pleurisy and diaphragmatic hernia formation, with a possible subcapsular liver abscess. In this hospitalization, the patient was not given oral anti-tuberculosis drugs because of obvious abdominal pain and abdominal distension after eating. The patient received intravenous infusions of 0.3 g isoniazid and 0.45 g rifampicin once daily for two weeks before surgical intervention. During the operation, a 5 × 5 × 3 cm^3^ encapsulated empyema was identified on the right mediastinal surface, containing extensive caseous necrotic tissue. The empyema had ruptured and flowed downward to the cardiophrenic angle, forming an encapsulated abscess. The abscess subsequently perforated the diaphragm, resulting in a diaphragmatic rupture measuring approximately 3 × 4 cm^2^. Extensive caseous necrosis was observed in the subcapsular liver abscess, but the liver parenchyma remained unaffected (Figure 2A). The encapsulated empyema was drained, the affected portion of the diaphragm was resected, the necrotic tissue beneath the liver capsule was excised, the chest cavity and liver-diaphragmatic surface were repeatedly disinfected with diluted povidone-iodine, and the diaphragmatic defect was repaired using a patch (Figure 2B). Postoperative pathology of necrotic tissue of the mediastinum and diaphragm indicated necrotizing granulomatous inflammation, and tuberculosis was considered (Figure 3). *Mycobacterium tuberculosis* was detected by Mtb-RNA test in necrotic tissue, but there was no tuberculosis growth in necrotic tissue by tuberculosis liquid culture and L-J solid culture. After the operation, the patient continued to receive oral HRZE anti-tuberculosis treatment. Following meals, the patient did not report any abdominal pain or bloating. Two weeks post-surgery, the patient showed improvement and was discharged. The patient was followed up by telephone for 5 months after discharge, and the symptoms of abdominal pain and abdominal distension did not recur.

## Discussion

Chronic empyema is characterized by prolonged purulent inflammation, pleural thickening, significant fibrosis, and necrotizing granulation tissue. In cases of chronic empyema, extensive adhesions within the thoracic cavity are observed, leading to an unclear boundary between lung tissue and the chest wall, thereby forming a closed pleural space. During the early stages of tuberculous empyema, neutrophil infiltration occurs but is subsequently replaced by macrophages, neutrophils and macrophages undergo destruction, releasing significant amounts of proteolytic enzymes. These enzymes dissolve fibrin tissue and thin the abscess wall, ultimately resulting in rupture. The pus from the empyema frequently spreads through the thoracic wall soft tissues and eventually penetrates the skin (8). However, diaphragmatic rupture caused by empyema is rarely reported. The early treatment of empyema can be through chest tube drainage, and injection of urokinase and other dissolved pus into the pleural cavity to promote the discharge of pus. When the empyema develops to the later stage, fibrous tissue hyperplasia causes pleural thickening and envelops the pus, which is difficult to drain through chest tube drainage. Surgery combined with drugs is an effective method for the treatment of encapsulated empyema.

The patient presents with tuberculous empyema located in the mediastinum, characterized by dense adhesions at the interlobar fissure and mediastinal surface. Chest CT revealed multiple enlarged and calcified lymph nodes in the mediastinum, as well as thickened and calcified pericardium. The empyema likely originated from lymph node tuberculosis infection, with subsequent spread following lymph node rupture, forming a gravitated abscess that is confined to the diaphragmatic angle. Following the destruction of neutrophils and macrophages, proteolytic enzymes were released in significant quantities, leading to diaphragmatic erosion. The patient’s primary complaint was right upper abdominal pain, which intensified postprandially, accompanied by increased abdominal pain and distension. Diaphragmatic hernia refers to the ectopic movement of abdominal parenchymal organs into the thoracic cavity through the diaphragm. The most common ones are esophageal hiatal hernia, thoracoabdominal hiatus hernia, parasternal hernia, etc. When diaphragmatic hernia causes gastroesophageal reflux, patients will have symptoms such as upper abdominal fullness, belching, and pain. Given the findings on the patient’s chest CT, misdiagnosis as a diaphragmatic hernia was plausible. During surgery, it was observed that the tuberculous abscess had invaded the subcapsular area of the liver without affecting the hepatic parenchyma. Preoperative transaminases and bilirubin levels showed no significant abnormalities. After clearance of the subcapsular abscess, the patient’s abdominal pain improved, indicating that the pain was primarily due to abscess compression of the liver. Studies have demonstrated that cases involving liver damage necessitate extensive resection of affected liver tissue, diaphragm, and surrounding normal tissues (9). Since no substantial damage to the hepatic parenchyma was noted in this patient, liver resection was not performed. After three months of postoperative follow-up, there was no recurrence of the subcapsular abscess. During surgery, the patient’s diaphragm was found to be thin and lacking contractile function. To prevent the recurrence of diaphragmatic rupture due to excessive tension after repair, we utilized a repair membrane for diaphragmatic reconstruction, which effectively prevented the recurrence of diaphragmatic rupture. To prevent tuberculosis progression and associated complications, oral HRZE anti-tuberculosis treatment was continued postoperatively.

Surgery in conjunction with drugs represents a critical approach for managing chronic tuberculous empyema. This method not only eradicates lesions that are inaccessible to pharmacological treatment but also effectively restrains the progression of tuberculosis. Tuberculous abscesses frequently rupture, resulting in the dissemination of the disease to adjacent tissues. Nevertheless, diaphragmatic rupture induced by this condition is relatively uncommon and may lead to misdiagnosis.

## Data Availability

The original contributions presented in the study are included in the article/supplementary material, further inquiries can be directed to the corresponding authors.

## References

[1] Yuanyuan L Jingjing X Shutao L Dongdong S Zheng T Lu P. 2024 WHO tuberculosis report: key data analysis for China and the global world. Elect J Emerg Infect Dis. (2024) 9:92–7.

[2] Colares PFB Rivas JKD Sciortino ADS Sales RKB Teixeira LR. Tuberculous empyema: combined intrapleural therapy might be an alternative. J Bras Pneumol. (2022) 48:e20220232. doi: 10.36416/1806-3756/e20220232, PMID: 36449818 PMC9747172

[3] Scarci M Abah U Solli P Page A Waller D van Schil P. EACTS expert consensus statement for surgical management of pleural empyema. Eur J Cardiothorac Surg. (2015) 48:642–53. doi: 10.1093/ejcts/ezv272, PMID: 26254467

[4] Davies HE Davies RJ Davies CW. Management of pleural infection in adults: British Thoracic Society pleural disease guideline 2010. Thorax. (2010) 65:ii41–53. doi: 10.1136/thx.2010.137000, PMID: 20696693

[5] Shaw JA Diacon AH Koegelenberg CFN. Tuberculous pleural effusion. Respirology. (2019) 24:962–71. doi: 10.1111/resp.13673, PMID: 31418985

[6] Rossi SE Erasmus JJ Mcadams PH. Thoracic manifestations of tuberculosis. Contemp Diagn Radiol. (2000) 23:1–8. doi: 10.1097/00219246-200023050-00001

[7] Gomes MM Alves M Correia JB Santos L. Empyema necessitans: very late complication of pulmonary tuberculosis. BMJ Case Rep. (2013) 2013:bcr2013202072. doi: 10.1136/bcr-2013-202072 PMC3863066 24326441

[8] Kohli S Agrohi J A GK Agrohi D. Protracted tuberculous empyema necessitans following intercostal drainage tube insertion. Int J Res Med Sci. (2023) 11:2722–5. doi: 10.18203/2320-6012.ijrms20232131

[9] Zhang L Han C Han Z Yang B Gao H Shi J. Two rare cases involving the spread of tuberculosis: A tuberculous abscess of the Chest Wall invading the liver by way of the diaphragm. Intern Med. (2016) 55:2237–9. doi: 10.2169/internalmedicine.55.5692, PMID: 27523001

